# Effect of antifungal triazoles on vinca alkaloid neurotoxicity in pediatric patients: A retrospective case series analysis and literature review

**DOI:** 10.1097/MD.0000000000047447

**Published:** 2026-01-30

**Authors:** Yinyu Zhao, Xiaolei Ren, Jiali Chen, Yidan Zhu, Nan Guo, Xiaohong Zhang, Lin Huang

**Affiliations:** a Department of Pharmacy, Peking University People’s Hospital, Beijing, China; b School of Pharmaceutical Sciences, Peking University, Beijing, China; c School of Clinical Pharmacy, Shenyang Pharmaceutical University, Shenyang, China.

**Keywords:** neurotoxicity, pediatric, triazoles, vinca alkaloids

## Abstract

To describe clinical manifestations, toxicities, and document measures for the management of neurotoxicity of vinca alkaloids (VAs) in pediatric cases. And discuss the difference between VAs monotherapy and combination of triazoles. A retrospective and descriptive analysis of adverse drug interaction (ADR) cases, which reported to the National Center for ADR monitoring from Peking University People’s Hospital from January 2010 to December 2023, was performed. We also herein present a review of the literature on neurotoxicity caused by VAs. One hundred twenty-three patients were included. Median age was 4 years (0.1–18 years) with 86 (70.7%) having a diagnosis of acute lymphoblastic leukemia. The median number of VAs doses administered after which the neurotoxicity occurred was 3 doses (1–12 doses). The median time to clinical manifestations of adverse events was 21 days (1–140 days). The most common neuropathic toxicity is autonomic neuropathy, with paralytic ileus accounting for the highest proportion. More patients experienced severe neurotoxicity when treated with the combination of triazoles and VAs than with VAs monotherapy. In clinical practice, doctors and clinical pharmacists should pay attention to this adverse drug interaction. It is recommended to monitor for signs of neurotoxicity and consider the use of alternative antifungal agents in patients receiving VAs chemotherapy, in order to mitigate the occurrence of this adverse drug interaction.

## 1. Introduction

Acute lymphoblastic leukemia (ALL) is the most common malignancy in childhood, of which 5-year overall survival (OS) rates exceeding 90%.^[1]^ Vinca alkaloids (VAs) are the earliest developed microtubule-targeting agents in the treatment of hematological and lymphatic neoplasms. And there are 5 VAs, including vincristine (VCR), vindesine (VDS), vinblastine, vinorelbine, and vinflunine, which are approved for clinical use.^[2]^ VCR and VDS are essential components of the combination chemotherapy of childhood ALL.^[3]^ Concerning the VAs metabolism, they are mediated by the cytochrome P450 CYP3A isoform and served as a substrate for the efflux transporter P-glycoprotein (P-gp).^[4]^

Due to the destruction of autoimmune function, the use of large amounts of immunosuppressive drugs, and the effects of chemotherapy, invasive fungal disease has become one of the most serious complications in patients with hematologic malignancies and an important cause of severe morbidity and high mortality.^[5]^ According to the latest guidelines, triazole antifungals (fluconazole, itraconazole, voriconazole, posaconazole, and isavuconazole), which are moderate or strong inhibitors of CYP3A4,^[6]^ are commonly used for the prevention and treatment of both Aspergillus and Candida infections.^[7-9]^ In addition, itraconazole and posaconazole would inhibit VAs transport by P-gp.^[4]^ From the metabolic mechanism of VAs and triazoles, it is clear that their combinations could lead to excess VAs exposure and the occurrence or aggravation of neurotoxicity.^[10]^

This adverse drug reaction (ADR) was first described by Murphy et al,^[11]^ they reported 5 children who used a combination of VCR and itraconazole and developed neurotoxicity. Since then, case reports and case series of this severe drug interaction continue to be reported. However, there are comparatively few cases reported in pediatric population and without a systematic summary. Our study retrospectively collected data from our single-center and systematically reviewed published case reports from both domestic and international sources, aiming to explore the effect of antifungal triazoles on vinca alkaloid neurotoxicity in pediatric patients. Our analysis includes demographic characteristics, clinical manifestations, toxicity profiles, and management strategies for adverse reactions between VAs and triazole drugs. This study is helpful in reminding clinicians to pay attention to the drug–drug interactions, enabling implementation of evidence-based preventive strategies and timely therapeutic interventions during pre-neurotoxic phases and post-manifestation scenarios.

## 2. Materials and methods

A retrospective and descriptive analysis of ADR cases, which reported to the National Center for ADR Monitoring from Peking University people’s hospital from January 2010 to December 2024, was performed. Those cases included in this study were patient’s age <18 years, only used VAs or coadministration of VAs and triazoles, occurrence of drug interactions about neurotoxicity during the course of treatment, such as paralytic ileus, posterior reversible encephalopathy syndrome (PRES) and syndrome of inappropriate antidiuretic hormone (SIADH), and all diagnoses were considered in this study. In contrast, the cases that adult, had no neurotoxicity were excluded. Written informed consent was obtained from all patients or legal representatives enrolled in the study.

We searched database Pubmed and Embase using “triazole, posaconazole, voriconazole, itraconazole, isavuconazole, fluconazole, vinca alkaloids, VAs, vincristine, vindesine, neurotoxicity, ADR, adverse drug reaction, case” as the keyword from the establishment of the database to December 2024. Inclusion criteria: patient’s age <18 years, only used VAs or coadministration of VAs and triazoles, case reports of VAs-related neurotoxicity; and case series studies. Exclusion criteria: adult; experimental studies; duplicates; poorly described or undescribed adverse effects; and adverse effects clearly not attributable to VAs.

Following details from each case are compiled and analyzed: age, sex, diagnosis, doses of VAs and triazoles, day of starting triazole treatment (number of days from onset of VAs therapy), interval to reaction (number of days to first appearance of adverse reaction from start of VAs therapy), time to recovery, treatment, fasting period and outcome. Data were extracted and organized using IBM SPSS Statistics version 27.0 (IBM Corp., Armonk) and subsequent statistical analyses were performed with the same software.

## 3. Results

A total of 123 patients were identified as meeting the case definition described above. The number of cases caused by coadministration of VAs and antifungal triazole agents in Peking University People’s Hospital and in the literature was 15 and 24, respectively. The number of cases caused by VAs alone in Peking University People’s Hospital and in the literature was 13 and 71, respectively. The specific details are summarized in Tables S1–S4, Supplemental Digital Content, https://links.lww.com/MD/R270. The demographics of these patients are summarized in Table 1. The median age for all patients was 4 years, ranging from 0.1 to 18. The primary diagnosis for most patients is ALL (70.0%). In Peking University people’s hospital, 15 interactions with VAs (VCR, n = 10, 66.7%; VDS, n = 5, 33.3%) were reported for the following triazoles antifungal agents: posaconazole (n = 9, 60.0%); voriconazole (n = 6, 40.0%). Cases reported in the literature, 24 interactions with VAs (VCR, n = 23, 95.8%; VDS, n = 1, 4.2%) were reported for the following triazoles antifungal agents: posaconazole (n = 4, 1.7%); itraconazole (n = 20, 98.3%). Of the patients taking VAs monotherapy (means VAs without triazoles), only 1 patient used VDS, and others used VCR. In total VAs + triazole cases, the median number of VAs doses administered after which the ADR occurred was 3 doses (range, 1–6 doses). The median time to clinical manifestations of adverse events was 18 days (range, 4–96 days). There was no mortality associated with the VAs and triazole antifungals drug interaction. All patients were recovered from these adverse drug interactions. In total VAs monotherapy cases, the median number of VAs doses administered after which the ADR occurred was 3 doses (range, 1–12 doses) and the median time to clinical manifestations of adverse events was 21 days (range, 1–140 days). Among the patients taking VAs monotherapy in literature, 64 patients (90.1%) were recovered, the remaining 1 patient became blind, 2 patients had persistent symptom and 4 patients died.

**Table 1:** Demographics of cases caused by coadministration of vinca alkaloids and antifungal triazole agents (Total = 123).

	Total	VAs + triazole	VAs + triazole in literature	Total VAs + triazole	VAs	VAs in literature	Total VAs
Number of cases	123	15	24	39	13	71	84
Age, median yr (range)	4 (0.1–18)	5 (1–11)	4 (1.25–16)	4 (1–16)	8 (2–16)	4 (0.1,18)	4 (0.1,18)
Male*⁄*female, N	77/36 (unknown 10)	6/9	10/5 (unknown 9)	16/14 (unknown 9)	10/3	51/19 (unknown 1)	61/22 (unknown 1)
Primary diagnosis							
All, N (%)	86 (70.0)	13 (86.6)	22 (91.7)	35 (89.7)	10 (76.9)	41 (57.7)	51 (60.7)
Others, N (%)	37 (30.0)	2 (13.4)	2 (8.3)	4 (10.3)	3 (23.1)	30 (42.3)	33 (39.3)
Type of VAs							
VCR, N (%)	114 (92.7)	10 (66.7)	23 (95.8)	33 (84.6)	12 (92.3)	69 (97.2)	81 (96.4)
VDS, N (%)	7 (5.7)	5 (33.3)	1 (4.2)	6 (15.4)	1 (7.7)	–	1 (1.2)
Type of triazole							
Posaconazole, N (%)	13 (33.3)	9 (60.0)	4 (1.7)	13 (33.3)	–	–	–
Voriconazole, N (%)	6 (15.4)	6 (40.0)	–	6 (15.4)	–	–	–
Itraconazole, N (%)	20 (51.3)	–	20 (98.3)	20 (51.3)	–	–	–
Number of vincristine doses given, median (range)*	3 (1–12)	4 (1–6)	2 (1–6)	3 (1–6)	2 (1–4)	3 (1–12)	3 (1–12)
Time to adverse drug interaction, median days (range)†	21 (1–140)	25 (4–46)	18 (7–96)	18 (4–96)	12 (2–26)	26 (1–140)	21 (1–140)
Resolution of adverse drug interaction							
Recovery, N (%)	116 (94.3)	15 (100.0)	24 (100.0)	39 (100.0)	13 (100.0)	64 (90.1)	77 (91.7)

ALL = acute lymphoblastic leukemia, BL = Burkitt’s Lymphoma, LBL = lymphoblastic lymphoma, VAs = vinca alkaloids, VCR = vincristine, VDS = vindesine.

* Number of vincristine doses given with the concomitant administration of antifungal azole after which adverse drug interaction occurred.

† Time to adverse drug interaction with the concomitant administration of vincristine and antifungal azole.

The major types of adverse drug interactions reported in cases receiving VAs are summarized in Table 2. They are divided into neuropathic toxicity which is categorized as autonomic, central, cranial and peripheral neuropathies. In pediatric patients the most common neurotoxicity in declining order included autonomic neuropathy (n = 114), central neuropathy (n = 71), cranial neuropathy (n = 66) and peripheral neuropathy (n = 47). The most common adverse effects included paralytic ileus in 33 patients (11.1%) and vocal cord palsy in 29 patients (9.7%), hypertension and constipation both occurred in 23 patients (7.7%). And SIADH in 22 patients (7.4%). Besides, PRES was only reported in cases from Peking University people’s hospital. Paralytic ileus (n = 20, 15.9%) is the most common ADR in total VAs + triazole cases. However, among all cases of VAs monotherapy, vocal cord palsy (n = 29, 16.9%) was the most common. Moreover, more signs and symptoms were observed in patients receiving VAs monotherapy than in those receiving coadministration of VAs and triazoles.

**Table 2:** Adverse events reported inn patients receiving vinca alkaloids and antifungal triazole agents.

Adverse event	Signs and symptoms	Total	VAs + triazole	VAs + triazole in literature	Total VAs + triazole	VAs	VAs in literature	Total VAs
Autonomic neuropathy	Hypertension	23 (7.7)	10 (22.7)	10 (12.2)	20 (15.9)	–	3 (2.0)	3 (1.7)
	Paralytic ileus	33 (11.1)	12 (27.3)	8 (9.8)	20 (15.9)	10 (52.6)	3 (2.0)	13 (7.6)
	Constipation	23 (7.7)	–	13 (15.9)	13 (10.3)	1 (5.3)	9 (5.9)	10 (5.8)
	Abdominal pain	18 (6.0)	1 (2.3)	13 (15.9)	14 (11.1)	–	4 (2.6)	4 (2.3)
	Stridor	3 (1.0)	–	–	–	–	3 (2.0)	3 (1.7)
	Vomiting	5 (1.7)	–	–	–	–	5 (3.3)	5 (2.9)
	Diarrhea	5 (1.7)	–	–	–	–	5 (3.3)	5 (2.9)
	Bowel/bladder dysfunction	1 (0.3)	–	–	–	–	1 (0.7)	1 (0.6)
	Neurogenic bladder	2 (0.7)	–	2 (2.4)	2 (1.6)	–	–	–
	Detrusor dysrunction	1 (0.3)	–	–	–	–	1 (0.7)	1 (0.6)
Central neuropathy	PRES	8 (2.7)	6 (13.6)	–	6 (4.8)	2 (10.5)	–	2 (1.2)
	SIADH	22 (7.4)	6 (13.6)	8 (9.8)	14 (11.1)	1 (5.3)	7 (4.6)	8 (4.7)
	Hyponatremia	10 (3.3)	3 (6.8)	7 (8.5)	10 (7.9)	–	–	–
	Seizure	13 (4.4)	2 (4.5)	7 (8.5)	9 (7.1)	–	4 (2.6)	4 (2.3)
	Headache	1 (0.3)	–	–	–	–	1 (0.7)	1 (0.6)
	Oral dyskinosia	1 (0.3)	–	–	–	–	1 (0.7)	1 (0.6)
	Blindness	10 (3.4)	–	–	–	–	10 (6.5)	10 (5.8)
	Blurry vision	1 (0.3)	–	–	–	–	1 (0.7)	1 (0.6)
	Choreic movement	1 (0.3)	–	–	–	–	1 (0.7)	1 (0.6)
	Limb tremor	4 (1.3)	–	–	–	1 (5.3)	3 (2.0)	4 (2.3)
Cranial neuropathy	Facial paralysis	3 (1.0)	–	–	–	–	3 (2.0)	3 (1.7)
	Hoarseness	3 (1.0)	–	–	–	–	3 (2.0)	3 (1.7)
	Sore throat	1 (0.3)	–	–	–	–	1 (0.7)	1 (0.6)
	VCP	29 (9.7)	–	–	–	–	29 (19.0)	29 (16.9)
	Dysphagia	5 (1.7)	–	–	–	–	5 (3.3)	5 (2.9)
	Squint	1 (0.3)	–	–	–	–	1 (0.7)	1 (0.6)
	Ptosis	15 (5.0)	–	4 (4.9)	4 (3.2)	1 (5.3)	10 (6.6)	11 (6.4)
	Jaw pain	9 (3.0)	–	1 (1.2)	1 (0.8)	–	8 (5.2)	8 (5.7)
Peripheral neuropathy	Muscle and bone pain	8 (2.7)	2 (4.5)	1 (1.2)	3 (2.4)	–	5 (3.3)	5 (2.9)
	Limb paralysis	8 (2.7)	2 (4.5)	3 (3.7)	5 (4.0)	2 (10.5)	1 (0.7)	3 (1.7)
	Abnormal gait	2 (0.7)	–	1 (1.2)	1 (0.8)	1 (5.3)	–	1 (0.6)
	Hyporeflexia	10 (3.4)	–	1 (1.2)	1 (0.8)	–	9 (5.9)	9 (5.2)
	Muscle weakness	5 (1.7)	–	1 (1.2)	1 (0.8)	–	4 (2.6)	4 (2.3)
	Muscle cramps	1 (0.3)	–	1 (1.2)	1 (0.8)	–	–	–
	Hypotonia	2 (0.7)	–	–	–	–	2 (1.3)	2 (1.2)
	Parasthesias	1 (0.3)	–	–	–	–	1 (0.7)	1 (0.6)
	Loss of sensation	1 (0.3)	–	–	–	–	1 (0.7)	1 (0.6)
	Foot drop	1 (0.3)	–	–	–	–	1 (0.7)	1 (0.6)
	Heel-cord tightness	1 (0.3)	–	–	–	–	1 (0.7)	1 (0.6)
	Asthenia	1 (0.3)	–	–	–	–	1 (0.7)	1 (0.6)
	inability to walk	6 (2.0)	–	1 (1.2)	1 (0.8)	–	5 (3.3)	5 (2.9)
Total		298 (100.0)	44 (100.0)	82 (100.0)	126 (100.0)	19 (100.0)	153 (100.0)	172 (100.0)

PRES = posterior reversible encephalopathy syndrome, SIADH = syndrome of inappropriate antidiuretic hormone, VCP = vocal cord palsy.

When neurotoxicity occurs with monotherapy of VAs or the combination of the 2 drugs, in addition to symptomatic treatment, such as fasting, fluid infusion and intravenous nutrition, most common managements were cessation of triazole antifungal drugs, cessation of VAs, the use of an alternative non-azole antifungal agents, or reduction the dosage of VAs or change of VAs types. Please refer to Table 3 for details. The most common management in patients on monotherapy was cessation of VAs (n = 41, 48.8%), and in patients on combination of VAs and triazoles it was cessation of VAs and triazoles (n = 11, 28.2%).

**Table 3:** Management of neurotoxicity.

Management	total	VAs + triazole	VAs + triazole in literature	Total VAs + triazole	VAs	VAs in literature	Total VAs
Unchanged of VAs	1 (0.8)	–	–	–	–	1 (1.4)	1 (1.2)
Reduction of the VAs	18 (14.6)	–	–	–	–	18 (25.4)	18 (21.4)
Change of VAs types	5 (4.1)	–	1 (4.2)	1 (2.6)	4 (30.8)	–	4 (4.8)
Change of VAs types, then reduction of the VAs dose	1 (0.8)	–	–	–	1 (7.7)	–	1 (1.2)
Change of VAs types, then cessation of VAs	2 (1.6)	–	–	–	1 (7.7)	1 (1.4)	2 (2.4)
Cessation of VAs	42 (34.1)	–	1 (4.2)	1 (2.6)	2 (15.4)	39 (54.9)	41 (48.8)
Unchanged of VAs + cessation of triazoles	3 (2.4)	–	3 (12.5)	3 (7.7)	–	–	–
Reduction of the VAs dose + cessation of triazoles	3 (2.4)	1 (6.7)	2 (8.3)	3 (7.7)	–	–	–
Change of VAs types + cessation of triazoles	5 (4.1)	5 (33.3)	–	5 (12.8)	–	–	–
Cessation of VAs + cessation of triazoles	11 (8.9)	9 (60.0)	2 (8.3)	11 (28.2)	–	–	–
Unknown + only symptomatic treatment	32 (26.0)	–	15 (62.5)	15 (38.5)	5 (38.4)	12 (16.9)	17 (20.2)
Total	123 (100.0)	15 (100.0)	24 (100.0)	39 (100.0)	13 (100.0)	71 (100.0)	84 (100.0)

VAs = vinca alkaloids.

## 4. Discussion

This analysis describes the demographics, toxicities and managements of 123 cases of monotherapy of VAs and concomitant use of VAs with triazoles antifungal agents. VAs, an alkaloid extracted from the periwinkle plant, Catharanthus roseus of the Apocynaceae family,^[2]^ is used in the treatment of ALL in both the induction and consolidation phases of the disease. VAs belong to the microtubule-targeting agent category,^[12]^ they inhibit cell mitosis through their binding to tubulin proteins in the mitotic spindle, and thus avoid its polymerization into microtubules.^[2]^ It is generally believed that its neurotoxicity is due to the destruction of microtubules in the axon, resulting in interference with axonal transport, which in turn causes axonal neuropathy. This neuropathy involves both sensory and motor fibers, but small sensory fibers are particularly susceptible. And the neurotoxicity is dose-related and cumulative with repeated dosage.^[13]^ The metabolism of VAs is mainly mediated by the CYP3A4 and the P-gp transporter. Triazole antifungal agents interact with VAs by inhibiting its metabolism through CYP3A4 and its transport by P-gp.^[14,15]^ Both itraconazole and posaconazole are inhibitors of CYP3A4 and P-gp.^[6,15]^ Fluconazole and voriconazole are inhibitors of CYP3A4, and neither 1 inhibit P-gp.^[6,15]^ That’s why their coadministration will increase the blood concentration of VAs, and then aggravate its neurotoxicity.^[16]^

Since 1980, 71 cases of neurotoxicity due to VAs monotherapy have been reported in pediatric patients^[17-49]^ (Table S4, Supplemental Digital Content, https://links.lww.com/MD/R270). And since 1995, there have been reports of cases of triazoles-related increased VAs neurotoxicity in pediatric patients^[11,50-59]^ (Table S2, Supplemental Digital Content, https://links.lww.com/MD/R270). According to previous studies, peripheral nerve injury is the most common manifestation of neurotoxicity,^[60]^ but autonomic neuropathy is the most common in our study. We presume that this discrepancy may be because peripheral neuropathy is common and mild, which is likely to be under-reported. Peripheral neuropathy including decreased tendon reflexes and limb sensory abnormalities, starting with decreased achilles tendon reflexes and abnormal fingertip sensations, respectively. The most common is the sensory abnormality of fingers and toes. In our research, patients have symptoms of muscle and bone pain, limb paralysis, parasthesias, etc. And in severe cases, abnormal gait, foot drop and inability to walk were observed. Cranial neuropathy usually involves the motor nerve, abducens nerve, trigeminal nerve, facial nerve, auditory nerve, etc, resulting in ptosis, diplopia, photophobia, jaw pain, facial paralysis, tinnitus and hoarseness, but the vagus nerve is seldom involved.^[61]^ Central neuropathy including cerebral dysfunction and cerebral axonal swelling,^[62]^ such as PRES and SIADH. Autonomic neuropathy may manifest as abdominal pain, constipation, vomiting, bowel/bladder dysfunction, and in severe cases, paralytic ileus.

In our study, the most frequent manifestation is paralytic ileus (33/298). Paralytic ileus obstruction is a clinical syndrome in which intestinal motility dysfunction leads to ineffective transit of intestinal contents.^[63]^ Its main manifestations are nausea, vomiting, abdominal distension, abdominal pain, accumulation of gas and fluid in the gastrointestinal tract, and reduced or delayed defecation.^[64]^ The neurotoxicity of VAs itself is the main factor in the development of paralytic ileus. After VAs chemotherapy, it affects the balance of the intestinal autonomic nervous system, or affects the local nerve conduction in the intestinal tract, so that intestinal peristalsis is weakened or disappeared.^[57]^ Paralytic ileus is more common in VAs + triazole combination cases than in VAs monotherapy cases. With other neurotoxicity except for paralytic ileus, SIADH and PRES is more severe. SIADH is a disorder of impaired water excretion caused by an inability to suppress secretion of antidiuretic hormone (ADH),^[65]^ and is among the most frequent causes of hyponatremia.^[66]^ The pathogenesis of VAs-induced SIADH is unclear. PRES is a clinicoradiological diagnosis and supported by magnetic resonance brain scan findings.^[67]^ The clinical manifestations of PRES in children are mainly seizures, headache, visual disturbances and impaired consciousness.^[68,69]^ The pathogenesis of PRES is mainly based on the cerebral hyperperfusion and dysfunction of the vascular endothelium.^[70-73]^ Rapid increase in blood pressure may lead to disruption of the cerebral blood flow barrier and vasogenic brain edema. Chemotherapeutic drugs, such as VAs, can damage vascular endothelial cells and destroy the blood–brain barrier. They both are conditions associated with the development of PRES.^[67,73]^ It can be found that there is more severe neurotoxicity occurs in total Vas + triazole cases, which may be due to increased VAs exposure caused by triazoles. The median time to an adverse drug interaction with triazoles was 21 days (range from 1 to 140) and to VAs monotherapy cases was 18 days (range from 4 to 96) The median number of VAs doses given after which the adverse drug interaction occurred with triazoles or without triazoles were both 3 doses. However, an adverse drug interaction may occur even after 1 dose of VAs administered simultaneously with an antifungal azole. In our VAs + triazole cases, there are 2 patients developed symptoms of ADR after 1 dose of VAs. One patient developed vomiting, constipation and abdominal pain on day 4 after 1 dose of VDS (1.8 mg) on day 1, and posaconazole oral suspension was started 8 days ago. Another patient developed symptoms on day 8 after 1 dose of VDS (2.2 mg) on day 1, and posaconazole oral suspension was lasted for about 4 to 5 months. Besides, Pekpak et al^[56]^ described a 14-year-old boy who developed paralytic ileus symptoms 7 days after receiving the combination of VCR and posaconazole. The impact of the VAs–triazoles interaction may occur when the triazole agent level reaches a steady state, which is typically 7 to 15 days after initiation of itraconazole,^[74]^ 5 days after initiation of voriconazole^[75]^ and 7 to 10 days after initiation of posaconazole.^[75]^ VAs induced neurotoxicity could be temporary and reversible, disappearing a few months after disuse of VAs according to the review of cases. Because the plasma half-lives of VCR and VDS are 23 to 85 hours and 20 to 24 hours, respectively.^[2]^

There is little information about risk factors for developing neurotoxicity during VAs treatment. The median age of patients in our study is 5 years (range, 1–11), and age appeared to be an adverse influence in neurotoxicity according to existing findings. Compared to adolescents and adults, younger children could tolerate higher dosage of VCR because of their greater and quicker ability to metabolize VCR and develop mild neurotoxicity.^[76]^ Apart from it, other risk factors of VAs-induced neurotoxicity including lymphoma, Caucasian population, diabetes, the number of chemotherapy cycles, VCR plasma concentration time curve, elevated serum alkaline phosphatase, patients with hereditary neuropathies especially Charcot–Marie–Tooth disease, coadministered with triazoles agents, and the CYP3A5*3 genotype.^[10,76,77]^ It is useful to develop an understanding of the risk factors, which will help reduce the incidence of adverse reactions. When patient is under the VAs chemotherapy, the doctor or pharmacist is suggested to advising he not to add triazoles without authorization, which would lead to ADR.

There are several approaches to managing neurotoxicity induced by VAs. Among all cases of VAs monotherapy, the management strategies, in decreasing order of frequency, were cessation of VAs, dose reduction of VAs, and switching to another type of VAs. It is an option to reduce the dosage of VAs or use other alternative VAs, but there is no clear answer as to how much to reduce the dosage. Usually we substitute VDS for VCR or decrease VAs by one-third in the next chemotherapy cycle.^[58]^ Seven patients in our monotherapy cases substituted VDS for VCR, and have not occurred similar adverse reactions. In total VAs + triazole cases, aside from adapting dosage and types of VAs, we could also adapt the usage of triazole. Discontinue the triazole antifungal drugs before initiating VAs chemotherapy or replace them with non-triazole antifungal drugs, such as echinocandin and amphotericin B. There were 2 patients each switched to caspofungin and micafungin in our VAs + triazole cases. And 1 patient was represented with amphotericin B. Adjusting the timing of triazoles administration is an another option. VCR follows a triphasic elimination after intravenous injection, with distribution, middle and terminal half-lives of 5 minutes, 2.3 hours, and 85 hours.^[78]^ Thus, after injection, VCR declines rapidly after an initial high concentration, followed by a prolonged, slow clearance at a lower concentration. Lin et al^[79]^ reported cases in which the administration of posaconazole was given on 48 hours prior and on the same day of VCR administration, and the neurotoxicity symptoms were reduced. Luer et al^[80]^ reported that posaconazole was stopped 48 hours before VCR chemotherapy and then added 24 hours later, resulting in a decrease in posaconazole concentration from >2 mg/L at the initial time to <0.5 mg/L, and no VCR-related neurotoxicity was observed in the children. It is also shown the importance of therapeutic drug monitoring of triazoles. Furthermore, it’s also important to note that some patients used nifedipine to lower blood pressure in our cases, which might have potential VAs-induced neurotoxicity. Nifedipine is a P-gp inhibitor and has been reported to decrease VCR plasma transport.^[4]^ Nifedipine need to be avoided in coadministered with VAs and triazoles.

This study has several limitations. First, as a single-center, small-sample retrospective analysis, the generalizability of its results may still be limited, although we have expanded the sample size to some extent by integrating cases from published literature. Second, retrospective data led to the absence of clinical information, preventing us from evaluating the inhibitory effects of triazoles on VAs under different doses and exposures. In addition, a significant constraint was the inability to perform standardized neurotoxicity grading for the adverse events. The reliance on chart documentation, without the opportunity for direct patient follow-up, may also have resulted in underreporting or incomplete capture of the full clinical presentation and severity of neurotoxicity. This limitation hindered a more nuanced analysis of toxicity severity and its correlation with potential risk factors.

## 5. Conclusion

We have reported and summarized 123 cases of VAs neurotoxicity in pediatric patients. The combination therapy with triazoles resulted in a higher number of patients with severer neurotoxicity caused by VAs, but had no clear effect on the number of VAs dosage given and time to adverse drug interaction. It is advisable to monitor for signs of neurotoxicity and consider the utilization of alternative antifungal agents in patients undergoing VAs chemotherapy, in order to mitigate the occurrence of this adverse drug interaction.

## Author contributions

**Conceptualization:** Yidan Zhu, Xiaohong Zhang, Lin Huang.

**Data curation:** Jiali Chen, Nan Guo.

**Supervision:** Xiaolei Ren.

**Writing – original draft:** Yinyu Zhao.

## Supplementary Material


