# Supplementary material for: Effect of antifungal triazoles on vinca alkaloid neurotoxicity in pediatric patients: A retrospective case series analysis and literature review
Source: Medicine (Baltimore). 2026 Jan 30;105(5):e47447. doi: 10.1097/MD.0000000000047447 (PMC12863776; doi:10.1097/MD.0000000000047447)
Supplement: Supplementary file 1 [file medi-105-e47447-s001.docx]

Supplementary Tables

Table S1. Summary of cases caused by coadministration of vinca alkaloids and antifungal triazole agents in our hospital. (Total=15)

| No. | Age(y) | sex | diagnosis | Adverse drug reactions | VAs (dosage) | Triazoles treatment (dosage)/ Administration route^*^ | Day of starting triazloe treatment^#^ | Interval to reaction | Treatment | Fasting Period | Outcome |
| --- | --- | --- | --- | --- | --- | --- | --- | --- | --- | --- | --- |
| 1 | 10 | M | ALL | Paralytic ileus, SIADH, Hypertension, PRES | VCR（1.5mg/m^2^ d1，d8，d15，d22） | Posaconazole (200mg tid) /oral | 45 | 46 | Fasting, fluid infusion, Mecobalamin, Captopril, Levetiracetam, Cessation of VCR and Posaconazole | 5d | Recovered |
| 2 | 1 | F | ALL | Paralytic ileus, Hypertension, hyponatremia | VCR（1.5mg/m^2^ d1，d8，d15， d22） | Posaconazole (200mg tid) /oral | - | 21 | Fasting, fluid infusion, Mecobalamin, Captopril, Cessation of VCR, Posaconazole replaced by micafungin | 9d | Recovered |
| 3 | 4 | F | ALL | Paralytic ileus, PRES, Hypertension | VDS（1.8mg d1） | Posaconazole (200mg tid) /oral | -8 | 4 | Fasting, fluid infusion, Tropisetron, Diazepam, intravenous nutrition, Nifedipine and Captopril, Cessation of VDS, Posaconazole replaced by micafungin and ornidazole | 4d | Recovered |
| 4 | 1 | M | ALL | Paralytic ileus, Hypertension | VCR（1.5mg/m^2^ d1，d8，d15， d22） | Posaconazole (200mg tid) /oral | 3 | 26 | Fasting, fluid infusion, intravenous nutrition, glycerol enema, gastrointestinal decompression, Mecobalamin, Captopril, Cessation of VCR and posaconazole | 5d | Recovered |
| 5 | 3 | F | ALL | Paralytic ileus, Hypertension, Muscle and bone pain | VDS（1.7mg d1，d8，d15） | Posaconazole (200mg tid) /oral | - | 27 | Fasting, fluid infusion, intravenous nutrition, Glycerine enema, Vitamin B12, Cessation of VDS and posaconazole, Nifedipine | 10d | Recovered |
| 6 | 3 | F | ALL | Paralytic ileus, SIADH, PRES, Hypertension | VCR（1.5mg/m^2^ d1，d8；0.8mg d15） | Posaconazole (200mg tid) /oral | 11 | 22 | Nifedipine and Captopril, Diazepam, Mannitol, Cessation of VCR and posaconazole | 11d | Recovered |
| 7 | 2 | M | ALL | SIADH, PRES, Hypertension | VCR（1.5mg/m^2^ d5，d12，d19，d26） | Voriconazole (7mg/kg bid) /iv | 28 | 34 | Phenobarbital, Diazepam, Mannitol, Voriconazole replaced by caspofungin, Captopril and metoprolol, VCR replaced by VDS (1mg) | - | Recovered |
| 8 | 5 | M | BL | Paralytic ileus, hyponatremia | VCR（1.5mg/m^2^ d1，1mg d8） | Posaconazole (200mg tid) /oral | 3 | 15 | Fasting, fluid infusion, Cessation of VCR, Posaconazole replaced by Amphotericin B | 4d | Recovered |
| 9 | 5 | M | ALL | Paralytic ileus, SIADH, PRES, Hypertension | VDS（2.2mg d1） | Posaconazole (200mg tid) /oral | -4~5months | 8 | Fasting, fluid infusion, Mannitol, Nifedipine and Captopril, Cessation of VCR and posaconazole | 7d | Recovered |
| 10 | 11 | F | ALL | Limb paralysis, Muscle and bone pain | VCR（1.5mg/m^2^ d2，d9；1.0mg/m^2^ d16，d23） | Voriconazole (7mg/kg bid) /oral | 26 | 30 | Mecobalamin, Cessation of Voriconazole, VCR replaced by VDS (3mg) | - | Recovered |
| 11 | 10 | F | ALL | Limb paralysis, Paralytic ileus, SIADH, Seizures, Hypertension | VCR（1.5mg/m^2^ d1，d8） | Posaconazole (200mg tid) /oral | 3 | 11 | Fasting, fluid infusion, intravenous nutrition, Mecobalamin, Diazepam, Mannitol, Nifedipine and Fosinopril, Levetiracetam, Posaconazole replaced by caspofungin, VCR replaced by VDS (1mg) | 9d | Recovered |
| 12 | 5 | F | LBL | Paralytic ileus, PRES, Hypertension, hyponatremia | VDS（1.8mg d1，d8，d15，d22，d29，d36） | Voriconazole (7mg/kg bid) /oral, then iv | 23 | 37 | Fasting, Nifedipine and Captopril, Mannitol, Phenobarbital, Voriconazole replaced by posaconazole, VDS reduced to 1.2mg | 9d | Recovered |
| 13 | 2 | F | ALL | SIADH, Seizures, Abdominal pain | VDS（1.8mg d1，d8，d15，d22，d29） | Voriconazole (7mg/kg bid) /oral | 23 | 30 | Mannitol, Phenobarbital, VDS replaced by VCR (0.9mg), Cessation of Voriconazole | - | Recovered |
| 14 | 7 | M | ALL | Paralytic ileus | VCR（1.5mg/m^2^ d1，d8，d15），VDS2.4mg d22 | Voriconazole (7mg/kg bid) /oral, then iv | 1 | 22 | Fasting, intravenous nutrition, Vitamin K1, Etamsylate, Cessation of VCR and Voriconazole | 6d | Recovered |
| 15 | 6 | F | ALL | Paralytic ileus | VCR（1.5mg/m^2^ d1，d8，d15，d22） | Voriconazole (7mg/kg bid) /oral | 22 | 25 | Fasting, fluid infusion, VCR replaced by VDS (2.5mg), Cessation of Voriconazole | 4d | Recovered |

^*^Number of days from onset of vinca alkaloid therapy.

^#^Number of days to first appearance of ileus from start of vinca alkaloid therapy.

M, male; F, female; d, day; LBL, Lymphoblastic lymphoma; ALL, acute lymphoblastic leukemia; BL, Burkitt's Lymphoma; SIADH, syndrome of inappropriate antidiuretic hormone; PRES, posterior reversible encephalopathy syndrome; VAs, vinca alkaloids; VCR, vincristine; VDS, vindesine

**Table S2**

Table S2. Review of cases caused by coadministration of vinca alkaloids and antifungal triazole agents reported in literature. (Total=24)

| Authors (year) | Age(y) | sex | diagnosis | Adverse drug reactions | VAs (dosage) | Triazoles treatment (dosage)/ Administration route^*^ | Day of starting triazloe treatment^#^ | Interval to reaction (day) | Treatment | Outcome |
| --- | --- | --- | --- | --- | --- | --- | --- | --- | --- | --- |
| Bohme1995^1^ | 16 | F | ALL | Paralytic ileus, Limb paralysis | VCR (2 mg every week) | Itraconazole (400 mg once daily)/Oral | 1 | 11 | Substituted with vinblastine | Recovered |
| Murphy1995^2^ | 11 | F | ALL | Constipation, Abdominal pain, Hypertension, SIADH | VCR (1.5 mg/m^2^ every week) | Itraconazole (2.5 mg/kg/ day)/Oral | 7 | 14 | - | Recovered |
| Murphy1995^2^ | 4 | M | ALL | Constipation, Abdominal pain, Hypertension, Hyponatremia | VCR (1.5 mg/m^2^ every week) | Itraconazole (2.5 mg/kg/ day)/Oral | 2 | 9 | - | Recovered |
| Murphy1995^2^ | 1.25 | M | ALL | Constipation, Abdominal pain, Hypertension, Seizures, Hyponatremia | VCR (1.5 mg/m^2^ every week) | Itraconazole (2.5 mg/kg/ day)/Oral | 3 | 18 | - | Recovered |
| Murphy1995^2^ | 8 | F | ALL | Constipation, Abdominal pain, Hypertension, Hyponatremia | VCR (1.5 mg/m^2^ every week) | Itraconazole (2.5 mg/kg/ day)/Oral | 7 | 17 | - | Recovered |
| Murphy1995^2^ | 2 | M | ALL | Constipation, Abdominal pain, Hypertension, Hyponatremia | VCR (1.5 mg/m^2^ every week) | Itraconazole (2.5 mg/kg/ day)/Oral | 6 | 16 | - | Recovered |
| Jeng2001^3^ | 5 | M | ALL | Paralytic ileus, Hyponatremia | VCR (1.5 mg/m^2^  every week) | Itraconazole (2.5 mg/kg/ day)/- | 1 | 21 | - | Recovered |
| Kamaluddin2001^4^ | 4 | - | ALL | Hypertension, SIADH | VCR (4.5 mg/m^2^ every week) | Itraconazole (5 mg/kg/ day)/Oral | 3 | 18 | - | Recovered |
| Kamaluddin2001^4^ | 4 | - | ALL | Constipation, Hyporeflexia | VCR (6 mg/m^2^ every week) | Itraconazole (5 mg/kg/ day)/Oral | 3 | 18 | - | Recovered |
| Kamaluddin2001^4^ | 6 | - | ALL | Abdominal pain, Constipation | VCR (6 mg/m^2^ every week) | Itraconazole (5 mg/kg/ day)/Oral | 4 | 25 | - | Recovered |
| Kamaluddin2001^4^ | 10 | - | ALL | Abdominal pain, Constipation, Hypertension, Seizures, SIADH | VCR (6 mg/m^2^ every week) | Itraconazole (5 mg/kg/ day)/Oral | 2 | 23 | - | Recovered |
| Kamaluddin2001^4^ | 3 | - | ALL | Abdominal pain, Constipation, Hypertension, SIADH | VCR (4.5 mg/m^2^ every week) | Itraconazole (5 mg/kg/ day)/Oral | 8 | 23 | - | Recovered |
| Kamaluddin2001^4^ | 3 | - | ALL | Ptosis, Abnormal gait | VCR (6 mg/m^2^ every week) | Itraconazole (5 mg/kg/ day)/Oral | 21 | 49 | - | Recovered |
| Kamaluddin2001^4^ | 3 | - | ALL | Abdominal pain, Constipation, Hypertension, Ptosis, Muscle weakness, SIADH | VCR (6 mg/m^2^ every week) | Itraconazole (5 mg/kg/ day)/Oral | 3 | 19 | - | Recovered |
| Kamaluddin2001^4^ | 14 | - | NHL | Abdominal pain, Constipation, Seizures, Muscle cramps | VCR (4.5 mg/m^2^ every week) | Itraconazole (5 mg/kg/ day)/Oral | 1 | 18 | - | Recovered |
| Kamaluddin2001^4^ | 4 | - | NHL | Abdominal pain, Seizures, Hypertension | VCR (6mg/m^2^ every week) | Itraconazole (5 mg/kg/ day)/Oral | 71 | 96 | - | Recovered |
| Ariffin2003^5^ | 8 | M | ALL | Ptosis, Paralytic ileus, Seizures, SIADH | VCR (1.5 mg/m^2^ every week) | Itraconazole (5 mg/kg/ day) /- | 1 | 18 | Cessation of itraconazole and reduction in dose of VCR | Recovered |
| Ariffin2003^5^ | 2 | M | ALL | Paralytic ileus, Inability to walk, Neurogenic bladder | VCR (1.5 mg/m^2^ every week) | Itraconazole (5 mg/kg/ day) /- | 1 | 8 | Cessation of VCR and itraconazole | Recovered |
| Bermudez2005^6^ | 3 | M | ALL | Paralytic ileus, Neurogenic bladder, Hyponatremia, Ptosis | VCR (1.5 mg/m^2^ every week) | Itraconazole (5 mg/kg/ day)/Oral | 7 | 17 | Cessation of VCR and itraconazole | Recovered |
| Eiden2009^7^ | 4 | F | ALL | Limb paralysis, Abdominal pain, Constipation, Seizures, SIADH | VCR 1.5 （mg/m^2^ on d2，d5) | Posaconazole (50mg 3 time a day)/Oral | 1 | 7 | Cessation of posaconazole | Recovered |
| Alavi2013^8^ | 8 | M | ALL | Jaw pain, Paralytic ileus | VCR (1.5 mg/m2 every week) | Posaconazole (-)/Oral | - | - | Cessation of posaconazole | Recovered |
| Pekpak2018^9^ | 14 | M | ALL | Muscle and bone pain, Paralytic ileus, Hyponatremia | VCR (-) | Posaconazole (-)/Oral | -14 | 7 | Cessation of posaconazole, Gabapentin | Recovered |
| Zhou2018^10^ | 4 | M | ALL | Abdominal pain, Constipation, Limb paralysis, Hypertension, Seizures, SIADH | VDS (3 mg/m2) every 4 weeks | Itraconazole (2.5 mg/kg, q12h) | 1 | 7 | Cessation of itraconazole ， fluid restriction and sodium supplementation，decreased dose of VDS (1 mg/m2) when co-administered with itraconzole | Recovered |
| Li2023^11^ | 17 | F | ALL | Paralytic ileus | VCR (2 mg on d1) d5: VDS (4 mg evert week) | Posaconazole (200 mg three times daily)/Oral | 2 | 11 | Substituted with intravenous caspofungin; cessation of VDS; glycerol enema, fasting, gastrointestinal decompression, parenteral nutrition; Da-Cheng-Qi Decoction for enema catharsis | Recovered |

^*^Number of days from onset of vinca alkaloid therapy.

^#^Number of days to first appearance of ileus from start of vinca alkaloid therapy.

M, male; F, female; d, day; ALL, acute lymphoblastic leukemia; NHL, non-Hodgkin’s lymphoma; SIADH, syndrome of inappropriate antidiuretic hormone; VAs, vinca alkaloids; VCR, vincristine; VDS, vindesine

1. Bohme A, Ganser A, Hoelzer D. Aggravation of vincristine-induced neurotoxicity by itraconazole in the treatment of adult ALL. *Ann Hematol* 1995; **71**(6): 311-2.

2. Murphy JA, Ross LM, Gibson BE. Vincristine toxicity in five children with acute lymphoblastic leukaemia. *Lancet* 1995; **346**(8972): 443.

3. Jeng MR, Feusner J. Itraconazole-enhanced vincristine neurotoxicity in a child with acute lymphoblastic leukemia. *Pediatr Hematol Oncol* 2001; **18**(2): 137-42.

4. Kamaluddin M, McNally P, Breatnach F, et al. Potentiation of vincristine toxicity by itraconazole in children with lymphoid malignancies. *Acta Paediatr* 2001; **90**(10): 1204-7.

5. Ariffin H, Omar KZ, Ang EL, Shekhar K. Severe vincristine neurotoxicity with concomitant use of itraconazole. *J Paediatr Child Health* 2003; **39**(8): 638-9.

6. Bermudez M, Fuster JL, Llinares E, Galera A, Gonzalez C. Itraconazole-related increased vincristine neurotoxicity: case report and review of literature. *J Pediatr Hematol Oncol* 2005; **27**(7): 389-92.

7. Eiden C, Palenzuela G, Hillaire-Buys D, et al. Posaconazole-increased vincristine neurotoxicity in a child: a case report. *J Pediatr Hematol Oncol* 2009; **31**(4): 292-5.

8. Alavi S, Ebadi M. Prolonged vincristine toxicity induced by concurrent posaconazole in a child with T-cell acute lymphoblastic leukemia. *Iran J Med Sci* 2013; **38**(2): 135-6.

9. Pekpak E, Ileri T, Ince E, Ertem M, Uysal Z. Toxicity of Vincristine Combined With Posaconazole in Children With Acute Lymphoblastic Leukemia. *J Pediatr Hematol Oncol* 2018; **40**(5): e309-e10.

10. Zhou H, Li L, Zhou Y, Han Y. Syndrome of inappropriate antidiuretic hormone secretion from concomitant use of itraconazole and vindesine. *J Clin Pharm Ther* 2018; **43**(1): 137-40.

11. Li Y, Gong YH, Zhao MF, Xiao X, Wei XC. Ileus induced by the combination of vinca alkaloids and posaconazole in a patient with acute lymphoblastic leukemia: a case report and literature review. *J Int Med Res* 2023; **51**(8): 3000605231193823.

**Table S3. Summary of cases caused by vinca alkaloids in our hospital. (Total=13)**

| No. | Age(y) | sex | diagnosis | Adverse drug reactions | VAs (dosage) | Interval to reaction (day) | Treatment | Fasting Period | Outcome |
| --- | --- | --- | --- | --- | --- | --- | --- | --- | --- |
| 1 | 3 | F | ALL | Paralytic ileus | VCR (1.5 mg/m^2^ d1, d8) | 12 | Fasting, fluid infusion, Mecobalamin, VCR replaced by VDS (1.5mg) | 5d | Recovered |
| 2 | 3 | M | ALL | Paralytic ileus, ptosis, abnormal gait | VDS (2 mg d1) | 3 | Fasting, fluid infusion, glycerol enema, cessation of VDS | 3d | Recovered |
| 3 | 2 | F | ALL | Paralytic ileus | VCR (1.5 mg/m^2^ d1, d8) | 10 | Fasting, fluid infusion, glycerol enema, VCR replaced by VDS (0.6mg) | 4d | Recovered |
| 4 | 8 | F | ALL | Paralytic ileus, Limb tremor | VCR (1.5 mg/m^2^ d1, d8) | 14 | Fasting, fluid infusion, Mecobalamin, glycerol enema, Vitamin B12 cessation of VCR | 4d | Recovered |
| 5 | 15 | M | ALL | Paralytic ileus, PRES | VCR (1 mg/m^2^ d1, d8) | 14 | Fasting, fluid infusion, VCR replaced by VDS, then VDS dose reduced | - | Recovered |
| 6 | 8 | M | ALL | Paralytic ileus | VCR (1.5 mg/m^2^ d1) | 26 | Fasting, fluid infusion, VCR replaced by VDS then cessated | 10d | Recovered |
| 7 | 13 | M | T-LBL/ALL | Paralytic ileus | VCR (1.5 mg/m^2^ d8, d15, d22, d29) | 3 | Fasting, fluid infusion, Mecobalamin, VCR replaced by VDS(3.9mg) | 7d | Recovered |
| 8 | 11 | M | ALL | Abdominal pain, Limb paralysis | VCR (1.5 mg/m^2^ d1) | 22 | Fasting, fluid infusion, VCR replaced by VDS(4.8mg) | - | Recovered |
| 9 | 5 | M | BL | Paralytic ileus | VCR (1 mg/m^2^ d1) | 17 | Fasting, fluid infusion | 8d | Recovered |
| 10 | 4 | M | ALL | Paralytic ileus | VCR (1.5 mg/m^2^ d1) | 8 | Fasting, fluid infusion, Mecobalamin | 14d | Recovered |
| 11 | 4 | M | ALL | Paralytic ileus | VCR (1.5 mg/m^2^ d1, d8, d15, d22) | 8 | Fasting, fluid infusion | 2d | Recovered |
| 12 | 8 | M | ALL | PRES | VCR (1.5 mg/m^2^ d1) | 20 | Levetiracetam, Valproic acid | - | Recovered |
| 13 | 16 | M | HL | SIADH, Limb paralysis | VCR (1 mg/m^2^ d1) | 2 | Fasting, fluid infusion, Mecobalamin | - | Recovered |

M, male; F, female; d, day; ALL, acute lymphoblastic leukemia; BL, Burkitt's Lymphoma; T-LBL/ALL, T lymphoblastic lymphoma/ leukemia; HL, Hodgkin's lymphoma; SIADH, syndrome of inappropriate antidiuretic hormone; PRES, posterior reversible encephalopathy syndrome; VAs, vinca alkaloids; VCR, vincristine; VDS, vindesine

**Table S4**

Table S4. Review of cases caused by vinca alkaloids reported in literature. (Total=71)

| Authors (year) | Age(y) | sex | diagnosis | Adverse drug reactions | VAs (dosage) | Interval to reaction (day) | Time to recovery | Treatment | Outcome |
| --- | --- | --- | --- | --- | --- | --- | --- | --- | --- |
| Awidi1980^12^ | 18 | M | Diffuse lymphocytic lymphoma | Blindness | VCR (2 mg) | 35 | - | Cessation of VCR | Blindness |
| Byrd1981^13^ | 7 | M | Burkitt’s lymphoma | Muscle and bone pain, Blindness, Seizure, Paralytic ileus, Atonic bladder | VCR (2 mg/m^2^ one day) | 6 | 12w | Cessation of VCR | Recovered |
| Byrd1981^13^ | 3.9 | M | Rhabdomyosarcoma | Hypertension, Paralytic ileus, Atonic bladder, Headache, Blindness, SIADH | VCR (1.8 mg/m^2^ every week) | 26 | - | Cessation of VCR | Recovered |
| Byrd1981^13^ | 11 | F | NHL | Jaw pain, Blindness, Seizure, SIADH | VCR (1.5 mg/m^2^ every week) | 2 | 50d | VCR regular 2 courses, then Cessation of VCR, fluids, antibiotics, cardiotonic drugs, and respiratory support | Recovered |
| Shurin1982^14^ | 15 | F | Medulloblastoma | Blindness, Hypertension, SIADH | VCR(-） | - | - | Cessation of VCR | Recovered |
| Tomiwa1983^15^ | 0.5 | M | Skin leukemia | SIADH, Choreic movement, Limb tremor, Oral dyskinesia, Seizure | VCR(0.4mg） | 5 | - | 3% sodium chloride solution | Died |
| Teichmann and Dabbagh1988^16^ | 11 | F | Astrocytoma | Blindness | VCR (1.5 mg/m^2^ every week) | 42 | - | Cessation of VCR | Died |
| McCuire1898^17^ | 2.5 | M | ALL | Mmuscle weakness | VCR (1.5 mg/m^2^ every week) | 34 | 11w | VCR 50% reduced | Recovered |
| Tobias and Bozeman1991^18^ | 5 | M | ALL | Sore throat, Muscle and bone pain, Stridor; VCP | VCR (1.5 mg/m^2^ every week) | 30 | Duration of Paralysis：3d | Cessation of VCR | Recovered |
| Tobias and Bozeman1991^18^ | 2 | F | T-cell lymphoma | Hypotonia, Parasthesiae, Constipation, VCP | VCR (1.5 mg/m^2^ every week) | - | 2w | VCR 50% reduced | Recovered |
| Annino1992^19^ | 1.5 | M | Embryonal rhabdomyosarcoma | Stridor, Vomiting, VCP | VCR(-） | - | Clinical resolution at 2w, Confirmed by DL at 6w | Cessation of VCR | Recovered |
| Annino1992^19^ | 1.3 | M | Brainstem ependymoma | Stridor, VCP | VCR(-） | - | 2w | Cessation of VCR | Recovered |
| Annino1992^19^ | 3 | M | ALL | Constipation, Hoarseness, VCP | VCR(-） | - | Clinical resolution at 2w | VCR given at half dose after normal DL | Recovered |
| Annino1992^19^ | 14 | F | NHL | Hoarseness, Facial paralysis, Blurry vision, Muscle and bone pain | VCR(-） | 28 | 4w | VCR full dose | Recovered |
| Graf1996^20^ | 9 | F | ALL | Muscle weakness, Hyporeflexia | VCR (1.5 mg/m^2^ every week) | 21 | 34w | - | Recovered |
| Stones1998^21^ | - | M | Rhabdomyosarcoma of the palate | Paralytic ileus, SIADH, Hypertension | VCR（9mg/m^2^）given in 6 days | 3 | Blood sodium recovery at 15d | intravenous fluids, antihypertensives, anticonvulsants, and antibiotics | Recovered |
| Anghelescu2002^22^ | 0.4 | - | ALL | Dysphagia, VCP | VCR four weekly (dose 1, 1mg/m^2^; doses 2 through 4, 1.125mg/m^2^） | 30 | 1w via DL | PPV for 1w, morphine and midazolam, dexamethasone, racemic epinephrine | Recovered |
| Schouten2003^23^ | 8 | M | NHL | Blindness | VCR (2 mg/m^2^ every week) | 16 | 4w | Cessation of VCR | Recovered |
| Müller2004^24^ | 2 | M | Wilms’ tumor | Ptosis | VCR (1.5 mg/m^2^ every week) | 49 | 7d | VCR full dose, Pyridoxine 300mg/m^2^/day Pyridostigmine 6mg/kg/day | Recovered |
| Duman2005^25^ | 2 | M | Sarcoma | Facial paralysis | VCR (1.4 mg/m^2^ every week) | 55 | 5d | Pyridoxine 150mg/m^2^/day | Recovered |
| Bay2006^26^ | 5 | F | ALL | Ptosis | - | 33 | 7d | Cessation of VCR, Pyridoxine 300mg/m^2^/day Pyridostigmine 6mg/kg/day | Recovered |
| Ozyurek2007^27^ | 4 | M | ALL | Ptosis | VCR (1.5 mg/m^2^ every week) | 21 | 14d | Pyridoxine 150mg/m^2^/day Pyridostigmine 3mg/kg/day | Recovered |
| Weisfeld-adams2007^28^ | 6 | M | Neuroectodermal tumor | Blindness | VCR (1.5 mg/m^2^ every week) | - | 48w | Cessation of VCR | Recovered |
| Ahmad2007^29^ | 3 | M | ALL | VCP | VCR (1.5 mg/m^2^ every week) | 21 | 38w(via laryngobrochoscopy) | Cessation of VCR | Recovered |
| Ahmad2007^29^ | 1.8 | M | Posterior fossa anaplastic ependymoma | Hoarseness, VCP | VCR (1.5 mg/m^2^ every week) | 35 | 34w | Cessation of VCR | Recovered |
| Ahmad2007^29^ | 2 | M | ALL | Hypotonia, Muscle weakness, VCP | VCR (1.5 mg/m^2^ every week) | 28 | VCR withheld temporarily until normal DL (at 26w) | Recurrence of symptoms with introduction of VCR subsequent Cessation of VCR | Recovered |
| Citak2008^30^ | 10 | M | Parameningeal alveolar rhabdomyosarcoma | Muscle weakness, Hyporeflexia, Foot drop, Constipation, Detrusor dysfunction | - | 87 | 12w | Terazosin 2 mg/d, Cessation of VCR | Recovered |
| Dejan2009^31^ | 5 | M | ALL | Ptosis | VCR (1.5 mg/m2 every week) | 26 | 14d | Pyridoxine 150mg/m^2^/day Pyridostigmine 3mg/kg/day | Recovered |
| Kuruvilla2009^32^ | 1 | F | ALL | Dysphagia, VCP | VCR (1.5 mg/m2 every week) | - | 37w | Cessation of VCR | Recovered |
| Kuruvilla2009^32^ | 4 | M | ALL | Jaw pain, Dysphagia, VCP | VCR (1.5 mg/m2 every week) | - | 20w | VCR recommenced at half dose | Recovered |
| Kuruvilla2009^32^ | 5 | M | Ewing sarcoma | VCP | VCR (1.5 mg/m2 every week) | - | 7w | course of VCR already completed at time of VCP diagnosis | Died |
| Kuruvilla2009^32^ | 3 | M | Rhabdomyosarcoma of the testes | Dysphagia, VCP | VCR (1.5 mg/m^2^ every week) | - | 4w | VCR recommenced at half dose, then increased to full dose | Recovered |
| Naithani2009^33^ | 14 | M | ALL | VCP | VCR (1.5 mg/m^2^ every week) | 38 | 35d | Cessation of VCR | Recovered |
| Latiff2010^34^ | 3 | F | ALL | VCP | VCR (1.5 mg/m^2^ every week) | 29 | - | Nebulized budesonide, debridement of the granulation tissue, unilateral laser posterior cordectomy, VCR were reduced by 50% | Recovered |
| Latiff2010^34^ | 2.5 | M | ALL | VCP | VCR (1.5 mg/m^2^ every week) | 18 | - | nebulized budesonide, VCR were reduced by 50% | Recovered |
| Baker2010^35^ | 46d | M | ALL | Ptosis, Facial paralysis, Limb paralysis | VCR (1.5 mg/m^2^ every week) | 16 | 40d | evocarnitine (LC) 150 mg tid, N-acetylcysteine (NAC) 50 mg bid, and pyridoxine 35mg daily | Recovered |
| Diezi2010^36^ | 3 | F | ALL | Diarrhea, Vomiting, Abdominal pain, Asthenia, Hyporeflexia | Cumulative VCR (21 mg/m^2^ ) | 63w | 12w | Cessation of VCR | Recovered |
| Diezi2010^36^ | 2.7 | M | ALL | Diarrhea, Vomiting, Abdominal pain, SIADH | VCR (days 1, 8, and 15 at 1.5 mg/m^2^ and day 22 at 2 mg/m^2^) | 23 | - | Cessation of VCR for 6 cycles , then full dose | Died |
| Diezi2010^36^ | 4 | F | Wilms’ tumor | Vomiting, Diarrhea | Cumulative VCR (15.2 mg/m^2^ ) | 28 | - | Cessation of VCR | Recovered |
| Diezi2010^36^ | 11 | M | ALL | Abdominal pain, Diarrhea | VCR (1.5 mg/m^2^ every week) | 8 | - | Cessation of VCR, then were reduced by 50%, then full dose | Recovered |
| Diezi2010^36^ | 14 | M | Hodgkin lymphoma | Abdominal pain, Diarrhea, Vomiting, SIADH | Cumulative VCR (3 mg/m^2^ ) | 12 | - | VCR was omitted on day 15 and VCR doses of days 1, 8, and 15 of the second OEPA cycle were administered at a 50% dose reduction. The first VCR dose was reduced by 50% and the second dose by 25%. Subsequent doses up to the end of treatment (6 doses) were given at 100% | Recovered |
| Gomber2010^37^ | 3.5 | M | ALL | Hyporeflexia, Muscle and bone pain | VCR (1.4 mg/m^2^ every week) | 14 | 24w | Cessation of VCR till recovery, then VCR 50% reduced | Recovered |
| Gomber2010^37^ | 6 | M | ALL | Hyporeflexia | VCR (1.4 mg/m^2^ every week) | 49 | 2w | Cessation of VCR till recovery, then VCR 50% reduced | Recovered |
| Gomber2010^37^ | 12 | M | ALL | Jaw pain | VCR (1.4 mg/m^2^ every week) | 1 | - | - | Recovered |
| Gomber2010^37^ | 12 | M | ALL | Jaw pain, Hyporeflexia | VCR (1.4 mg/m^2^ every week) | 1 | 3w | Cessation of VCR till recovery | Recovered |
| Gomber2010^37^ | 2 | F | ALL | Hyporeflexia | VCR (1.4 mg/m^2^ every week) | 14 | - | Cessation of VCR | Recovered |
| Gomber2010^37^ | 5 | M | NHL | Constipation, Seizure, Blindness | VCR (1.4 mg/m^2^ every week) | 14 | 1w | Cessation of VCR till recovery, then VCR 50% reduced | Recovered |
| Gomber2010^37^ | 11 | M | NHL | Jaw pain | VCR (1.4 mg/m^2^ every week) | 1 | - | - | Recovered |
| Gomber2010^37^ | 12 | M | NHL | Dysphagia, Hyporeflexia | VCR (1.4 mg/m^2^ every week) | 70 | - | Cessation of VCR | Recovered |
| Gomber2010^37^ | 12 | M | NL | Jaw pain, Loss of sensation | VCR (1.4 mg/m^2^ every week) | 14 | 2w | Cessation of VCR till recovery | Recovered |
| Gomber2010^37^ | 10 | M | NBL | Jaw pain | VCR (1.4 mg/m^2^ every week) | 1 | - | - | Recovered |
| Farruggia2012^38^ | 1.5 | F | ALL | VCP | VCR (1.5 mg/m^2^ every week) | 28 | 28d | VCR 1/3reduced | Recovered |
| Talebian2014^39^ | 2.5 | M | Wilms’ tumor | Ptosis | VCR (0.067mg/kg/d) | 21 | 7d | pyridoxine (150 mg/m^2^ po BID), and pyridostigmine (3 mg/kg po BID) | Recovered |
| Palkar2015^40^ | 2 | M | ALL | Ptosis, Muscle weakness | VCR(all 2.8 mg） | 35 | 4w | Cessation of VCR, pyridoxine (40 mg orally twice daily) and pyridostigmine (3 mg/kg/day) | Recovered |
| Zavala2019^41^ | 17 | F | ALL | VCP, Constipation | VCR (1.5 mg/m^2^ every week) | 15 | 112d | VCR 50% reduced, then regular | Recovered |
| Zavala2019^41^ | 3 | M | ALL | VCP, Ptosis, Jaw pain | VCR (1.5 mg/m^2^ every week) | 14 | 28d | VCR 50% reduced | Recovered |
| Zavala2019^41^ | 4 | M | ALL | VCP, Muscle and bone pain, Hyporeflexia, Heel-cord tightness, Muscle weakness | VCR (1.5 mg/m^2^ every week) | 21 | Still paretic after 3.3 years | VCR 1/10th dose, then omitted | Recovered |
| Tay2021^42^ | 1.5 | M | HB | VCP | VCR (1.5 mg/m^2^ every week) | 49 | 25d | Racemic epinephrine, nebulized levalbuterol, and intranasal oxygen.VCR 50% reduced, then omitted | Recovered |
| Tay2021^42^ | 2 | M | HB | VCP | VCR (1.5 mg/m^2^ every week) | 35 | 56d | Cessation of VCR | Recovered |
| Godbehere2021^43^ | 2.5 | F | ALL | VCP | VCR(-） | 21 | - | Omitted x2 VCR doses induction 50% doses in IM escalated to 75% and in DI No VCR in maintenance. | Recovered |
| Godbehere2021^43^ | 2.25 | F | ALL | VCP, Ptosis, Constipation | VCR(-） | 28 | - | Cessation of VCR | Recovered |
| Godbehere2021^43^ | 4 | M | ALL | VCP | VCR(-） | 98 | - | Cessation of VCR | Recovered |
| Godbehere2021^43^ | 2.8 | F | ALL | VCP | VCR(-） | 28 | - | Omitted x1 VCR doses induction and no doses in IM Full doses during DI No VCR in maintenance | Recovered |
| Godbehere2021^43^ | 2.8 | M | ALL | VCP | VCR(-） | 14 | - | Omitted x3 VCR doses induction Given HD MTX for IM Vinblastine during DI No VCR in maintenance. | Recovered |
| Godbehere2021^43^ | 2 | M | ALL | VCP, Constipation | VCR(-） | 28 | - | Omitted x1 VCR doses induction 50% doses in DI No VCR in maintenance | Recovered |
| Godbehere2021^43^ | 4 | M | ALL | VCP, Ptosis, Squint | VCR(-） | 140 | - | Changed to Reg A IM. 50% doses in DI No VCR in maintenance. | Recovered |
| Egan-Sherry2021^44^ | 11.6 | F | ALL | Inability to walk, Inability to use hands, Blindness, VCP Constipation | VCR (1.5 mg/m^2^ every week) | - | - | Cessation of VCR | Recovered |
| Egan-Sherry2021^44^ | 12.6 | F | ALL | Inability to walk, Limb tremor, Constipation | VCR (1.5 mg/m^2^ every week) | - | - | Switched from VCR to vinblastine，then Cessation of vinblastine， | Recovered |
| Egan-Sherry2021^44^ | 12.7 | M | ALL | Inability to walk | VCR (1.5 mg/m^2^ every week) | - | - | Cessation of VCR | persistent symptom |
| Egan-Sherry2021^44^ | 3 | M | ALL | Inability to walk | VCR (1.5 mg/m^2^ every week) | - | - | Cessation of VCR | Recovered |
| Egan-Sherry2021^44^ | 10 | M | ALL | Inability to walk, Bowel/bladder dysfunction | VCR (1.5 mg/m^2^ every week) | - | - | Cessation of VCR | persistent symptom |

M, male; F, female; d, day; w, week; ALL, acute lymphoblastic leukemia; NHL, non-Hodgkin’s lymphoma; SIADH, syndrome of inappropriate antidiuretic hormone; VAs, vinca alkaloids; VCR, vincristine; VDS, vindesine; DL, direct laryngoscopy; ICU, intensive care unit; IPAP, intermittent positive airway pressure; PPV, positive pressure ventilation; VCP, vocal cord palsy; CMT, Charcot-Marie-Tooth; DS, Down syndrome; EP, ependymoma; ES, Ewing sarcoma; HB, hepatoblastoma; IPAP, intermittent positive airway pressure

1. Bohme A, Ganser A, Hoelzer D. Aggravation of vincristine-induced neurotoxicity by itraconazole in the treatment of adult ALL. *Ann Hematol* 1995; **71**(6): 311-2.

2. Murphy JA, Ross LM, Gibson BE. Vincristine toxicity in five children with acute lymphoblastic leukaemia. *Lancet* 1995; **346**(8972): 443.

3. Jeng MR, Feusner J. Itraconazole-enhanced vincristine neurotoxicity in a child with acute lymphoblastic leukemia. *Pediatr Hematol Oncol* 2001; **18**(2): 137-42.

4. Kamaluddin M, McNally P, Breatnach F, et al. Potentiation of vincristine toxicity by itraconazole in children with lymphoid malignancies. *Acta Paediatr* 2001; **90**(10): 1204-7.

5. Ariffin H, Omar KZ, Ang EL, Shekhar K. Severe vincristine neurotoxicity with concomitant use of itraconazole. *J Paediatr Child Health* 2003; **39**(8): 638-9.

6. Bermudez M, Fuster JL, Llinares E, Galera A, Gonzalez C. Itraconazole-related increased vincristine neurotoxicity: case report and review of literature. *J Pediatr Hematol Oncol* 2005; **27**(7): 389-92.

7. Eiden C, Palenzuela G, Hillaire-Buys D, et al. Posaconazole-increased vincristine neurotoxicity in a child: a case report. *J Pediatr Hematol Oncol* 2009; **31**(4): 292-5.

8. Alavi S, Ebadi M. Prolonged vincristine toxicity induced by concurrent posaconazole in a child with T-cell acute lymphoblastic leukemia. *Iran J Med Sci* 2013; **38**(2): 135-6.

9. Pekpak E, Ileri T, Ince E, Ertem M, Uysal Z. Toxicity of Vincristine Combined With Posaconazole in Children With Acute Lymphoblastic Leukemia. *J Pediatr Hematol Oncol* 2018; **40**(5): e309-e10.

10. Zhou H, Li L, Zhou Y, Han Y. Syndrome of inappropriate antidiuretic hormone secretion from concomitant use of itraconazole and vindesine. *J Clin Pharm Ther* 2018; **43**(1): 137-40.

11. Li Y, Gong YH, Zhao MF, Xiao X, Wei XC. Ileus induced by the combination of vinca alkaloids and posaconazole in a patient with acute lymphoblastic leukemia: a case report and literature review. *J Int Med Res* 2023; **51**(8): 3000605231193823.

12. Awidi AS. Blindness and vincristine. *Ann Intern Med* 1980; **93**(5): 781.

13. Byrd RL, Rohrbaugh TM, Raney RB, Jr., Norris DG. Transient cortical blindness secondary to vincristine therapy in childhood malignancies. *Cancer* 1981; **47**(1): 37-40.

14. Shurin SB, Rekate HL, Annable W. Optic atrophy induced by vincristine. *Pediatrics* 1982; **70**(2): 288-91.

15. Tomiwa K, Mikawa H, Hazama F, et al. Syndrome of inappropriate secretion of antidiuretic hormone caused by vincristine therapy: a case report of the neuropathology. *J Neurol* 1983; **229**(4): 267-72.

16. Teichmann KD, Dabbagh N. Severe visual loss after a single dose of vincristine in a patient with spinal cord astrocytoma. *J Ocul Pharmacol* 1988; **4**(2): 117-21.

17. McGuire SA, Gospe SM, Jr., Dahl G. Acute vincristine neurotoxicity in the presence of hereditary motor and sensory neuropathy type I. *Med Pediatr Oncol* 1989; **17**(6): 520-3.

18. Tobias JD, Bozeman PM. Vincristine-induced recurrent laryngeal nerve paralysis in children. *Intensive Care Med* 1991; **17**(5): 304-5.

19. Annino DJ, Jr., MacArthur CJ, Friedman EM. Vincristine-induced recurrent laryngeal nerve paralysis. *Laryngoscope* 1992; **102**(11): 1260-2.

20. Graf WD, Chance PF, Lensch MW, Eng LJ, Lipe HP, Bird TD. Severe vincristine neuropathy in Charcot-Marie-Tooth disease type 1A. *Cancer* 1996; **77**(7): 1356-62.

21. Stones DK. "Vincristine overdosage in paediatric patients". *Med Pediatr Oncol* 1998; **30**(3): 193.

22. Anghelescu DL, De Armendi AJ, Thompson JW, Sillos EM, Pui CH, Sandlund JT. Vincristine-induced vocal cord paralysis in an infant. *Paediatr Anaesth* 2002; **12**(2): 168-70.

23. Schouten D, de Graaf SS, Verrips A. Transient cortical blindness following vincristine therapy. *Med Pediatr Oncol* 2003; **41**(5): 470.

24. Muller L, Kramm CM, Tenenbaum T, Wessalowski R, Gobel U. Treatment of vincristine-induced bilateral ptosis with pyridoxine and pyridostigmine. *Pediatr Blood Cancer* 2004; **42**(3): 287-8.

25. Duman O, Tezcan G, Hazar V. Treatment of vincristine-induced cranial polyneuropathy. *J Pediatr Hematol Oncol* 2005; **27**(4): 241-2.

26. Bay A, Yilmaz C, Yilmaz N, Oner AF. Vincristine induced cranial polyneuropathy. *Indian J Pediatr* 2006; **73**(6): 531-3.

27. Ozyurek H, Turker H, Akbalik M, Bayrak AO, Ince H, Duru F. Pyridoxine and pyridostigmine treatment in vincristine-induced neuropathy. *Pediatr Hematol Oncol* 2007; **24**(6): 447-52.

28. Weisfeld-Adams JD, Dutton GN, Murphy DM. Vincristine sulfate as a possible cause of optic neuropathy. *Pediatr Blood Cancer* 2007; **48**(2): 238-40.

29. Ahmed A, Williams D, Nicholson J. Vincristine-induced bilateral vocal cord paralysis in children. *Pediatr Blood Cancer* 2007; **48**(2): 248.

30. Citak EC, Oguz A, Karadeniz C, et al. Vincristine-induced peripheral neuropathy and urinary bladder paralysis in a child with rhabdomyosarcoma. *J Pediatr Hematol Oncol* 2008; **30**(1): 61-2.

31. Dejan S, Dragana B, Ivana P, Borivoje B, Marko P. Vincristine induced unilateral ptosis. *J Pediatr Hematol Oncol* 2009; **31**(6): 463.

32. Kuruvilla G, Perry S, Wilson B, El-Hakim H. The natural history of vincristine-induced laryngeal paralysis in children. *Arch Otolaryngol Head Neck Surg* 2009; **135**(1): 101-5.

33. Naithani R, Dolai TK, Kumar R. Bilateral vocal cord paralysis following treatment with vincristine. *Indian Pediatr* 2009; **46**(1): 68-9.

34. Latiff ZA, Kamal NA, Jahendran J, et al. Vincristine-induced vocal cord palsy: case report and review of the literature. *J Pediatr Hematol Oncol* 2010; **32**(5): 407-10.

35. Baker SK, Lipson DM. Vincristine-induced peripheral neuropathy in a neonate with congenital acute lymphoblastic leukemia. *J Pediatr Hematol Oncol* 2010; **32**(3): e114-7.

36. Diezi M, Nydegger A, Di Paolo ER, Kuchler H, Beck-Popovic M. Vincristine and intestinal pseudo-obstruction in children: report of 5 cases, literature review, and suggested management. *J Pediatr Hematol Oncol* 2010; **32**(4): e126-30.

37. Gomber S, Dewan P, Chhonker D. Vincristine induced neurotoxicity in cancer patients. *Indian J Pediatr* 2010; **77**(1): 97-100.

38. Farruggia P, Tropia S, Cannella S, Bruno G, Oddo G, D'Angelo P. Vocal cord palsy after vincristine treatment in a child and the inefficacy of glutamic acid in the prevention of relapse: a case report. *J Med Case Rep* 2012; **6**: 128.

39. Talebian A, Goudarzi RM, Mohammadzadeh M, Mirzadeh AS. Vincristine-induced cranial neuropathy. *Iran J Child Neurol* 2014; **8**(1): 66-8.

40. Palkar AH, Nair AG, Desai RJ, Potdar NA, Shinde CA. Vincristine-Induced Neuropathy Presenting as Ptosis and Ophthalmoplegia in a 2-Year-Old Boy. *J Pediatr Ophthalmol Strabismus* 2015; **52 Online**: e34-7.

41. Zavala H, Roby BB, Day A, Bostrom B, Sidman J, Chinnadurai S. Vincristine-induced vocal cord paresis and paralysis in children. *Int J Pediatr Otorhinolaryngol* 2019; **123**: 1-4.

42. Tay SY, Foster J, Heczey A, Sitton M. Pediatric Oncology Patients With Vincristine-Induced Recurrent Laryngeal Nerve Palsy: Two Case Reports and a Brief Review of Literature. *Ear Nose Throat J* 2021; **100**(10): NP459-NP63.

43. Godbehere J, Payne J, Thevasagayam R. Vocal cord paralysis secondary to vincristine treatment in children: A case series of seven children and literature review. *Clin Otolaryngol* 2021; **46**(5): 1114-8.

44. Egan-Sherry D, Bhuta R, Cole PD, et al. Severe Vincristine-related Neurotoxicity in 5 Patients With Pediatric Acute Lymphoblastic Leukemia Requiring Discontinuation of Vincristine: A Description of Long-term Outcome. *J Pediatr Hematol Oncol* 2021; **43**(7): e997-e9.
